# Economics of Sesame and Its Use Dynamics in Ethiopia

**DOI:** 10.1155/2022/1263079

**Published:** 2022-08-30

**Authors:** Teshome Sirany, Esubalew Tadele

**Affiliations:** ^1^Department of Rural Development, Debre Markos University, Debre Marqos, Ethiopia; ^2^Department of Agricultural Economics, Debre Markos University, Debre Marqos, Ethiopia

## Abstract

Ethiopia's oilseed industry makes a major contribution to foreign exchange revenues. Ethiopia's three main oilseed crops (sesame, soybean, and Niger seed) account for about 20% of the country's total agricultural export profits, second only to coffee. Even though Ethiopia is one of the world's largest producers and exporters of sesame seeds, the country is facing increasing supply and demand restrictions. This paper begins with an examination of one of the most prominent oil crops in the country. It is a highly adaptable crop that may be used for anything from subsistence to commercial output. We established a comprehensive scientific understanding of the crop using a systematic review of the current literature and deductive logical reasoning that can be used to inform future research and policies. Various exclusion and inclusion criteria were used to filter the most notable findings. Millions of growers and other market participants are employed throughout the oilseed value chain. Reduced sesame productivity, pests and diseases, and limited access to modern technologies are all severe supply-side constraints. On the demand side, traders and market distortion, as well as an artificially higher home price and the ease with which unskilled labor can enter the market, are all factors. Other demand-side constraints include worldwide price volatility, a highly concentrated export market, and intense global competition. Ethiopia's sesame seed development potential is being severely hampered by these restrictions. If farmers, dealers, and the government do not address these issues strategically, the country may soon lose its competitiveness in the global sesame seed market. This will contribute to Ethiopia's ongoing discussion about how to better inform private and public sector policies and investments to increase sesame production, transform agriculture, improve nutrition and food systems, and be able to ease supply- and demand-side restrictions. In a nutshell, an increased area under cultivation combined with best agronomic practices could boost sesame production. Farmers, policymakers, researchers, and other stakeholders must thus intervene to enhance sesame production. Future studies should concentrate on how to boost sesame output in farmers' fields while following appropriate sesame production technology and agronomic principles.

## 1. Introduction

Sesame, the world's oldest oil crop, has adapted to the tropics and subtropics all around the world. Ethiopians have produced the crop for both oil and seed for millennia, well beyond Abyssinia's founding in the late first century B.C. Sesame, after coffee, is the second most significant agricultural product in terms of foreign exchange earnings, with about 449 million USD [1, 2].

In Asia, sesame oil is a high-quality food oil with a distinct flavor and odor [3]. Sesame seed oil is abundant in free fatty acids, primarily linoleic acid (37–47 percent), oleic acid (35–43 percent), palmitic (9–11 percent), and stearic acid (5–10 percent), containing trace levels of linolenic acid [4]. According to Queiroga et al. [5], a score of 3.22 is regarded as acceptable. Gouveia et al. [6] revealed that crude sesame oil has nutritive value since it contains more polyunsaturated fatty acids than saturated fatty acids.

For example, we are more familiar with canola oil, olive oil, and even avocado oils. Sesame oil, then again, is a popular cooking oil in Chinese, Japanese, and Middle Eastern cuisines. Raw or roasted sesame seeds are used to make sesame oil. Raw sesame oils are light in color and have a smooth, neutral taste. Toasted types, on the other hand, are darker, richer, and more creamy in flavor. Both have a wide range of cooking purposes. Sesame oil is commonly used in the preparation of meats and vegetables, as well as in dressings and marinades.

Sesame oil contains 82% free fatty acids [7]. It is especially rich in omega-6 fatty acids. Omega-6 fatty acids are a form of polyunsaturated fat that is vital to your diet and helps to avoid heart disease. Sesame oil quality is connected to the presence of bioactive chemicals, which might vary depending on the extraction procedure. According to Xu et al. [8] and Esmaeilzadeh et al. [9], sesame oil is found in total phenolic content, flavonoids, and pigments and has good antioxidant action. These findings suggest that supplementation with it was advantageous due to the presence of high quantities of essential polyphenolic components and that it may be utilized as a suitable functional food additive.

Sesame oil has excellent antioxidants. It includes lignans, sesamol, and sesaminol, in addition to vitamin E and phytosterols [10, 11]. These elements assist your body in fighting free radicals, which may lower your chances of acquiring chronic diseases. Sesame oil has a healthy combination of omega-3, omega-6, and omega-9 fatty acids. Polyunsaturated fatty acids are omega-3 and omega-6, whereas monounsaturated fatty acids are omega-9. According to the research findings, eating sesame oil reduces your chances of getting heart disease [12].

Sesame is a multipurpose crop that grows both cultivated and wild in Ethiopia, with great variation in cultivated sesame (*Sesamum indicum* L.). Sesame, also known as “Selit” and “white gold,” is a cash crop with a multibillion-dollar business that supports the livelihoods of thousands of small farmers, medium-to-large-scale private farms, and thousands of other players participating in the production-to-consumption/export chain. Domestic and foreign markets are drawn to the sesame crop [13]. This indicates that it is the backbone of the rural agricultural society and plays a critical role in the national economy.

Sesame is an orphan crop, despite its importance, because it has received little support from science, industry, or policymakers. As a result, it lags behind the other main oilseed crops in terms of genetic advancement [14]. Seed cracking, unpredictable growth habits, and asynchronous capsule ripening are still present in cultivated sesame, resulting in a low seed yield (300–400 kg/ha) [15]. Furthermore, sesame is frequently cultivated in hard conditions and is subjected to a variety of biotic and abiotic stressors, all of which significantly reduce its yield [16]. As a result, increasing sesame output and seed quality has become critical to meeting the growing demand for sesame oil.

## 2. Review Methods

To ensure thorough article retrieval, this review article is based on systematic review procedures and a thorough search of empirical findings across numerous databases. The authors' experience and long-term exposure to people who have long-term sesame farming were also considered during the review process. This review is also organized by temporal and spatial factors that might filter information, such as current and previous studies that show the dynamic function of sesame crops. The authors attempted to comprehend scientific data from a range of studies to create user dynamics that connect how to produce, process, transport, buy, consume, and dispose of food.

Furthermore, the authors respond to the following questions to provide the reader with a complete picture of the suggested crop to combat hunger and food insecurity and to try to exploit the dynamics of sesame, as well as popular oil crops, nutrition potential, health benefits, economic roles, and social and industrial outcomes of the sesame food system.

### 2.1. Search Engine Strategies

This review paper utilizes data from Google Scholar, Web of Science, Scopus, MPDI, and SAGE, with an emphasis on peer-reviewed journal articles, books, reports, journals, and conferences. Keywords like “Sesame,” “food system,” “Sesame contribution,” “Sesame farming,” “Sesame nutritional content,” and “present challenges of sesame production in Ethiopia” were used to narrow down the results. Over 110 journal articles, books, conference papers, thesis works, and government reports were searched for this review, with more than 70 materials being prioritized for use.

### 2.2. Spatial Production Distribution of Sesame

Because of the growing awareness of sesame's nutritional and health advantages, the consumption of sesame seeds and oil has been steadily rising. Sesame, on the other hand, is ideally suited to replace low-yield crops due to its low irrigation requirements, adaptability to various types of soil and weather conditions, lack of labor intensity, and high remuneration, particularly in the present context of global warming influencing crop productivity in increasingly conventional agricultural areas [14].

**Figure 1:**
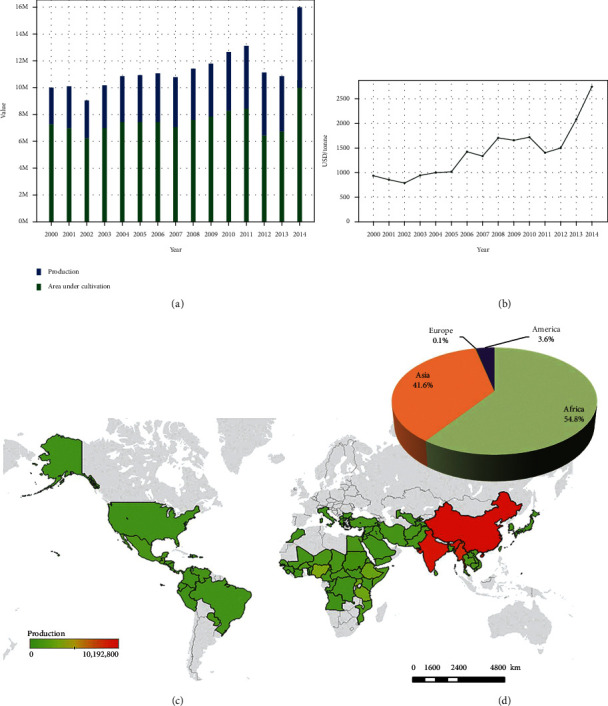
Sesame seed production in the world. (a) Evolution of sesame seed production and area under cultivation from 2000 to 2014. (b) Evolution of sales. Prices of sesame seed from 2000 to 2014. (c) Production share of sesame seed by continent in 2014. (d) Map of production quantities of sesame seed by country. Based on cumulative data from 2000 to 2014 (source: Food and Agriculture Organization Statistical Databases [17]).

As a result, sesame production has risen rapidly in recent years, and it is now an economically important crop for smallholders, assisting in the alleviation of rural poverty (Figure 1). Sesame was ranked ninth among some of the top oil crops in 2014, with over 6 million tons of seeds produced on over 11 million hectares (Food and Agriculture Organization Statistical Databases) [17].

**Figure 2:**
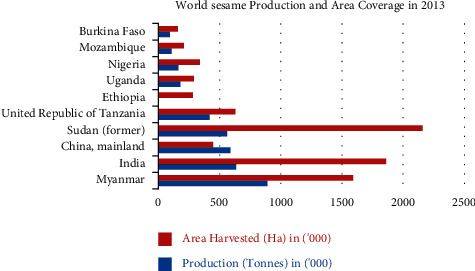
World sesame production and area coverage in 2013.

The total agricultural area accounts for 5.87 percent of the total global output. Sudan, India, Myanmar, the United Republic of Tanzania, China, and the mainland are the key leading nations in terms of sesame product harvested area, whereas Myanmar, India, and China are the top producers of sesame in the globe (Figure 2).

**Figure 3:**
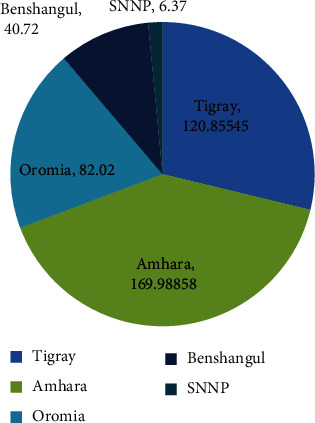
Sesame production by region in Ethiopia, 2014/15. Source: data from [1].

Sesame is grown in the Amhara, Tigray, Oromia, Southern Nations, Nationalities, and People's Region, Benishangul-Gumuz, and Somali regions of Ethiopia (Figure 3). Oilseed farming provides a living for more than 3.7 million smallholders [18]. Ethiopia's most significant oilseed crop is sesame. It grows from sea level to 1500 meters above sea level, with consistently distributed rainfall of 500 to 800 mm and temperatures ranging from 20 to 30 degrees Celsius, depending on the soil conditions [19].

Sesame seed is one of the most frequently grown oilseed crops in the country, accounting for 30% of the total oilseed output. The majority of production occurs in Ethiopia's northern and northwestern regions, which border Sudan and Eritrea. According to data from the Ministry of Trade and Industry (MoTI), Amhara accounts for 44% of national sesame seed output, followed by Tigray (31%) and Oromia (13%). The Benishangul-Gumuz, SNNPR, and Gambela areas account for 9%, 2%, and 1% of total output, respectively.

Sesame, the white gold crop, is the second most exported crop in the world after coffee, accounting for 14% of total global exports. Sesame output in Ethiopia is expanding as a consequence of the commodity's importance in the lives of sesame-growing farmers as well as an increase in demand and price.

### 2.3. Production Potential of Sesame in Ethiopia

This white gold grows both cultivated and wild in the world, with great variation in the cultivated sesame. Because of its economic importance, its output and productivity are growing year after year, and it can provide a respectable yield even in dryland regions. As shown in Table 1, China, the mainland, the United Republic of Tanzania, Ethiopia, Uganda, Burkina Faso, and Myanmar produce the most kg/ha of sesame in the globe.

Ethiopia is a significant sesame producer and exporter in Africa, sending a large amount of sesame to the global market. It is a key crop in Amhara, Tigray, Oromia, Benishangul-Gumuz, and the Southern Nations, Nationalities, and People's Region (SNNPR), with productivity (ton/ha) (refer to Table 2).

Table 3 refers to comparisons of the productivity of sesame seeds with other oilseed crops for the years 2008 and 2013. In 2008–2013, it was also said that sesame oil crop production (ton/ha) was equivalent to other oil crops such as groundnut, linseed, and *noug.*

### 2.4. Estimated Production Volume of Major Oilseeds (Metric Tons)

Ethiopia is a key site of origin and diversification for a variety of oil crops. The country's main oil crops are sesame, Niger seed, and soybeans. Producers and businesses, industries, the national economy, and consumers all benefit from these crops in different ways.

The majority of production occurs in Ethiopia's northern and northwestern regions, which border Sudan and Eritrea. As can be seen from Table 4, in the estimated comparisons of major oilseeds, sesame takes the highest proportion change from the year 2019/20 to 2018/19.

### 2.5. Food System Map for Sesame Production in Ethiopia

A food system map is a representation of a system. The placement of graphics on the map helps to orient the viewer to what is going on. It permits the whole system to be seen at once, even if just peripherally, allowing patterns to be uncovered and detected across the system. Parts of the image may be inspected in detail while also being observed in connection with one another while viewing. As a result, it can serve as an “entry point” into a complicated system with several elements.

Writing down a primary subject and brainstorming fresh and related ideas radiating out from the center of a food system map. You may organize knowledge in a way that will help you better absorb and remember information by focusing on essential concepts put down in your own words and looking for links between them. It was also created as a useful approach for creating ideas through association. To make a food system map, begin in the center of the page with the primary theme/main idea and expand outward in all directions from there to construct a developing diagram made up of keywords, phrases, concepts, facts, and statistics.

**Figure 4:**
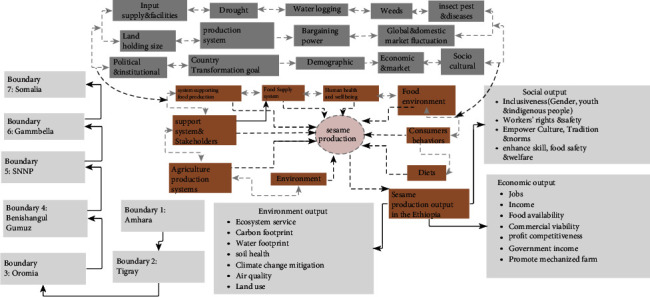
Food system map for sesame production in Ethiopia. Source: authors' own elaboration.

Figure 4 is a representation of a complex issue. It attempts to capture the real situation through a nonlinear pictorial of all ideas or concepts already addressed, linkages, links, impacts, cause-and-effect, and so on, centered on a primary concept or theme. As a result, the user can construct an intuitive framework around a primary notion. It may also transform a long list of dull data into a colorful, memorable, well-organized graph that follows your brain's natural workflow.

This food system map depicts the intricacy of sesame production through unstructured and nonlinear representations of the situation. It is used to help people reach higher levels of attention and creativity, as well as greater understanding and connection. This food system map might be a useful way to provide an overview of sesame production, enable a simpler representation of sesame production, and enable you to plan/make decisions about the resource material you have for once where you are going to do/produce this white gold crop. This encourages you to see the pathways of sesame production and use. It also guides the production boundaries, key constraints, drivers, and system interactions, as well as system sesame production and output (for details, refer to Figure 4).

### 2.6. Level of Influence for Sesame Production in Ethiopia

For investment, a key stakeholder is required. Anyone who is affected by the project or investment, including individuals, groups, and companies, can influence it. This person may have a direct or indirect interest in the project's work and may communicate with it on a daily or irregular basis. Stakeholders may come in a variety of shapes and sizes (e.g., local communities: families, employees, local political organizations, politicians, local and national NGOs, etc.).

Furthermore, the word “stakeholder” refers to people, groups, or organizations that have been actively involved in the project or whose interests may be affected positively or negatively because of the investment project's execution or successful completion. Everyone who has an interest in investing or with whom one needs to cooperate in some manner to accomplish the investment plan does not require the same level of attention.

We need to assess their degree of engagement and information demand now that we have tried to understand the stakeholders. A well-designed investment plan will not only identify important stakeholder responsibilities but also specify who participates to the greatest extent feasible. All stakeholders do not need to be involved in all parts of the project at all stages of its lifecycle. A person, group, or organization must have some level of interest, relevance, or influence over the production strategy to be called a stakeholder.

**Figure 5:**
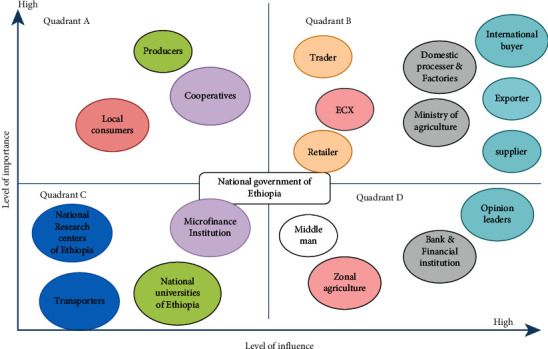
Stakeholder analysis using level of influence and importance for sesame production. Source: authors' own elaboration.

Figure 5 shows that a stakeholder's relative control over and within a production plan is shown by their impact. The extent to which the project cannot be regarded as successful if requirements, expectations, and difficulties are not handled is determined by its importance.

This stakeholder map depicts a client group's linkages, dependencies, interests, and power balances. The numerous dimensions of various stakeholder traits, including power, influence, interest, and attitude, are frequently represented using a matrix technique (Figure 5). It demonstrates that the level of influence of stockholders is high, while the level of influence of stockholders on the left is low, and the level of importance of participants in the bottom-up movement is high, while the level of importance of participants on the downside is low.

### 2.7. Catch Me If You Can: Sesame Trade

Among the most promising resources in the nation are coffee, legumes, oilseeds, cereals, potatoes, sugarcane, and vegetables. Ethiopia is one of the world's top six sesame producers. Sesame is one of the oldest oilseed crops, and it is produced in tropical and subtropical climes in Asia, Africa, and South America [21].

Sesame is the second most significant export crop after coffee, accounting for 14% of total world exports [22]. Ethiopia's global export contribution climbed from 1.5 percent in volume and 1.9 percent in value in 1997 to 8.9 percent and 8.3 percent in 2004 (CSA [23]). Ethiopian sesame producers rely heavily on sesame cultivation for living.

**Figure 6:**
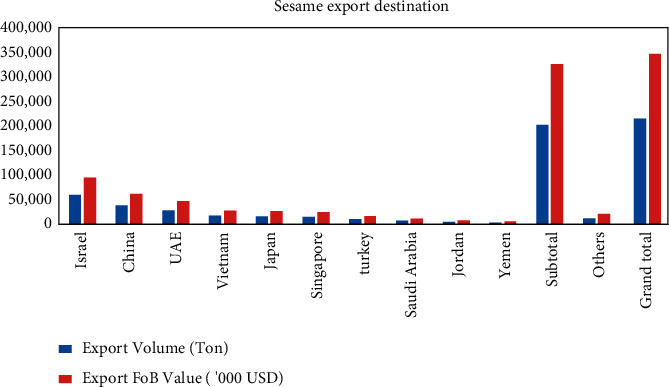
Sesame export destination.

Furthermore, it is mostly produced for export in Ethiopia, with just around 5% estimated to be consumed domestically [22]. Humera, Gondar, and Wollega sesame seeds are high-quality sesame that accounts for over 30% of the country's total sesame seed production [23]. Local farmers, investors, and farmer associations, as well as commercial and smallholder farms, are all interested. Ethiopia's largest export markets are now Israel, China, the United Arab Emirates, and Vietnam; refer to Table 5 and Figure 6.

According to the United States Department of Agriculture's Ethiopia Oilseeds 2020 annual report, the three principal oilseed crops (sesame, soybean, and Nigger seed) account for about 20% of Ethiopia's overall agricultural export revenues, second only to coffee.

**Figure 7:**
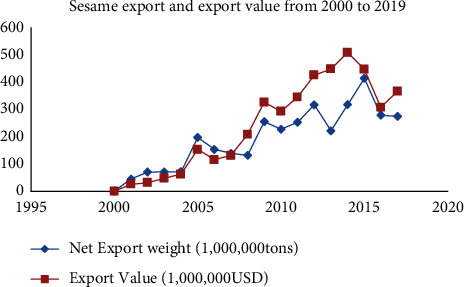
Sesame export and export value from 2000 to 2014. Source: data from [1], TDM (Trade Data Monitor (TDM) obtains the data from Ethiopian Customs Commission), and FAS Addis Ababa Forecast.

Figure 7 shows that Ethiopia exported 346,833 tons of sesame seeds for $509.505 in 2014. However, the value of sesame exports to other countries fell in 2015. Ethiopian sesame seed exporters buy raw sesame seed, hull it, crush it, grind it, and add value to it before reexporting it to Japan, Korea, Europe, and the United States [22]. It is critical to invest in value addition and postharvest operations to increase sesame export earnings. In the global market, sesame value-added products have more negotiating power and appeal.

The primary reasons for decreasing export trade performance/value, according to traders, include international price instability, currency fluctuations, excessive speculators, illicit trade, constricted productivity levels, and local market pricing distortions. The large gap between FOB export pricing and local trade prices of ECX exemplifies the distortion of the local market price.

### 2.8. Deep and Shallow Leverage Points of Sesame Production

Ethiopian provinces that cultivate sesame include Amhara, Tigray, Gambela, Benishangul-Gumuz, Somalia, SNNPR, and Oromia. Sesame, on the other hand, is exceedingly inefficient. This white golden crop contains both deep and shallow leverage points, which may limit yield.

**Figure 8:**
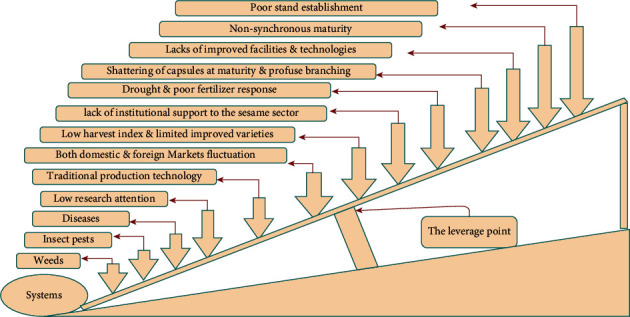
Leverage point of sesame production. Source: authors' own elaboration.

The invasion of desert locusts and unexpected showers in the country during and after the primary harvest collecting season are defensive predictors of sesame yield.  Figure 8 depicts a shallow and deep leverage point. When the lever (fulcrum) falls, the sesame production suffers greatly (severely). This demonstrates a greater need for action. As the lever is raised, the issues become less severe. The quality and quantity constraints on sesame production necessitate specific attention from the ground up.

Despite being one of the world's leading producers and exporters of sesame seeds, Ethiopia is increasingly facing supply- and demand-side constraints. A lack of access to modern technology, pests, and diseases are all examples of significant supply-side constraints.

On the demand side, asymmetrically higher domestic pricing, the ease with which novice merchants can enter the market, market distortion, and contractual nonperformance of export sales are other demand-side restrictions. These include international price volatility, a highly concentrated export market, and intense international rivalry. Ethiopia's sesame seed development potential is being threatened by these limits. If farmers, dealers, and the government do not handle these issues strategically, the country's competitiveness in the global sesame seed market may be endangered in the near future.

The following problems, in addition to weeds, insect pests, and illnesses, are considered barriers to sesame cultivation and poor yield in Ethiopia:

- Drought: in Ethiopia, the unexpected beginning and cessation of rainfall have an impact on sesame output, productivity, and quality. Despite the fact that sesame is a drought-tolerant crop, extended dry spells during its early stages of development have an impact on growth and development. Pollination and seed set are hampered when rainfall is scarce during the mid-growth phases (flower initiation and grain filling). El Nino affected sesame output in Ethiopia in 2015 [24], with full crop loss in the Humera area.
- Low sesame variety production: sesame is inherently a low yielder, but sesame variety productivity in Ethiopia is especially low when compared to other nations.
- Market volatility (domestic and world markets).
- Traditional production system: farmers and investors engage in poor management methods.
- Traditional plowing technique: farmers utilize a traditional plowing technique, such as oxen, donkey, or camel-drawn plow, which is labor-intensive and expensive to produce, has shallow till, and makes row planting and fertilizer application difficult.
- Less attention: sesame research receives less attention than other crops such as maize and wheat, despite the fact that it is a major export commodity, second only to coffee.
- Lack of modern machines: the majority of sesame growers are farmers who cannot afford modern planting, harvesting, and threshing machines.
- Lack of upgraded facilities.
- Poor sesame crop fertilizer reaction.
- Shattering: when natural sesame capsules attain maturity and harvesting is delayed, they shatter and drop seeds.
- Shattering: it causes a significant loss of sesame yield, even when harvested and bundled locally as “Hilla.”
- Shattering: a good solution is to collect the harvest on a smooth floor or on plastic sheets.
- Less productivity: sesame productivity is less than 10 qt/ha in most areas under the farmer's management.
- Vast production system: instead of intense production, investors utilize a vast production system.
- Traditional tillage tactics: traditional tillage tactics used by small-scale farmers in Ethiopia resulted in low sesame output and productivity, according to El Naim and Ahmed [25].
- Smallholder farmers' and cooperatives' low capacity and productivity, facilities such as hulling firms, and high inflation.

### 2.9. Use Dynamics of Sesame Product

**Figure 9:**
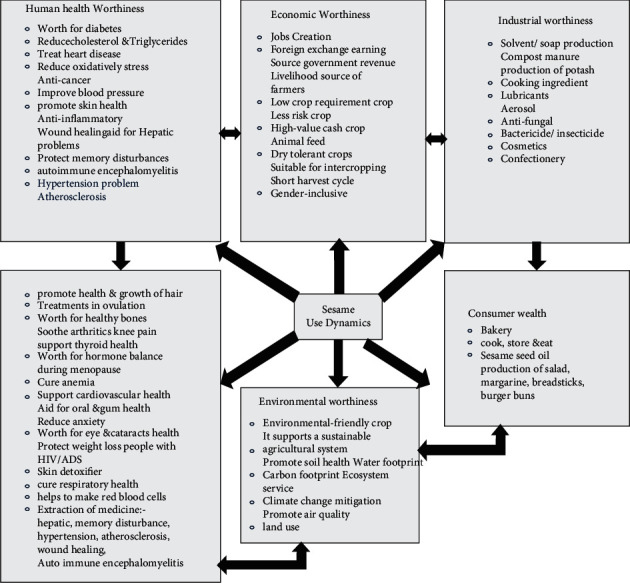
Sesame dynamics. Source: authors' own elaboration.

Sesame oil is regarded to be unique among oil crops, according to Figure 9. The product has a significantly longer shelf life and more stable features since natural antioxidants like sesame and *sesamolin*, as well as their derivatives (*sesamol* and *sesaminol*), are present. Sesame oil is mostly used in cooking. Soaps, paints, fragrances, medicines, and pesticides all include sesame oil. The cake that results from extracting oil from sesame seeds is a high-protein feed for poultry and ruminants [4].

Figure 9 illustrates that domestic demand for sesame is likely to rise, notably in the local food processing industry, as a result of the construction of integrated agroindustrial parks that will begin operations soon. Sesame hulling, roasting, and further processing and manufacture of other value-added goods are predicted to increase in these agroindustrial parks. On the other hand, sesame seeds' growing appeal as a key ingredient in a variety of cuisines, confectionery, and pharmaceutical and medicinal applications will fuel the rising global demand for sesame seeds.

This global trend will continue as consumer habits change as a result of rising health consciousness, an increase in vegans, and a growing desire for specialty foods like tahini, hummus, and snack bars, among other things. Demand is projected to rise in the coming years as other specialty segments that make sesame-based foods expand.

Figure 9 is explained in further detail. When used topically, sesame oil contains antioxidants that promote hair growth. It also possesses antiaging and skin-beneficial qualities. Because of its abundance of zinc, calcium, and phosphorus, it also possesses antibacterial characteristics that promote dental health and even boost bone health. It is also supposed to help males avoid infertility. Chemotherapy also protects DNA cells. Sesame seeds have a nutty taste and are commonly used in numerous cuisines such as spices and sauces. Many Indian foods include sesame seeds sprinkled on top to give nutritional benefits and a distinct flavor. They are also utilized to improve the flavor of baked goods. Sesame oil is a high-nutrient oil that is often used in cooking.

### 2.10. Opportunities and Values for Sesame Production in Ethiopia

**Figure 10:**
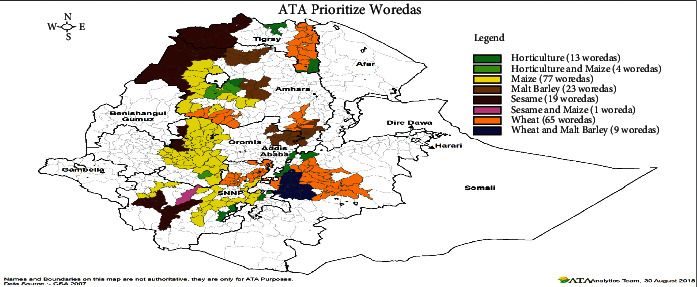
Crop cluster farming. Source: [26].

Sesame yields are predicted to rise in Ethiopia as well. A productive crop was aided by using improved seeds, additional inputs, and better farming systems such as row planting and improved agronomic methods. The predicted volume also considers the beneficial yield benefits of agricultural commercialization.  Figure 10 shows a cluster farming strategy that was recently established and used by ATA (Agricultural Transformation Agency). Sesame production is one of the key crops chosen as part of the cluster farming in order to boost its production and productivity as indicated by the blue shaded potentials of sesame production across different regions of Ethiopia whereby northern and northwestern parts of Ethiopia take the huge proportion of sesame production.

Ethiopia's government has sponsored integrated agroindustrial parks (IAIPS), which would provide new options to process the expected rise in oilseed output, implying that cooking oil exports may decline in the future. On the other hand, large regions for sesame farming, irrigable land, inexpensive labor, government support for the commodity, and varietal diversity are among the production potential for sesame.

These will play a critical role in providing modern sesame production systems in Ethiopia, necessitating improved production techniques, increased productivity, and the development of varieties with higher productivity potential, wider adaptability, and improved crop protection techniques, as well as capacity building for experts on agronomic practices.

Furthermore, Ethiopia's diverse agroecology is conducive to sesame cultivation. Ethiopia grows a variety of sesame cultivars. The following are the potential and prospects for sesame production in Ethiopia:

- In various regions of Ethiopia (most preferably Amhara, Benishangul-Gumuz, Asosa, Gambela, Oromia, Somalia, and SNNPR), there is a large area suitable for sesame production.
- Demand in the global market: Ethiopian sesame is exported to China, Israel, the United States of America, and Egypt.
- Regardless of the foreign cash, the crop has received less attention.
- There is a plentiful supply of workers at peak seasons (planting, weeding, and harvesting).
- There is financial institution expansion to fund sesame production.
- There is Increased irrigation availability throughout the country.
- Elamin et al. claim that the 2015 sesame crop can perform well under irrigation, with a water consumption efficiency of 1.8 to 1.6 m^3^/kg [1].

### 2.11. Nutritional Comparison of Sesame with Major Stable Crops

Food is a basic need that has evolved into a human right, as well as a source of energy, power, disease defense, and strength for all species to live and do their daily responsibilities. It is also critical for survival, and achieving it has a substantial influence on ongoing economic and other activities in humans as well as other livestock. The most common sources of vegetable oil include sesame, linseed, soybeans, maize, cottonseed, peanut, sunflower, and rapeseed. Sesame seed is a high-value food crop that is used as a spice as well as a source of edible oil (in bakeries).

Nutritional comparisons are made at various dates, locations, laboratories, formats, and researchers. It demonstrates that sesame products have higher nutritional value than some other oil crops; they include less of certain other food grains and are equivalent to the nutritional values of some other oil crops. Furthermore, the sesame crop is high in calories, fat, protein, calcium, phosphorus, iron, and ash, as well as other macro- and micronutrients (see details Table 6).

Furthermore, Ethiopian sesame oil includes a high concentration of fatty acids, namely, linoleic (39.3–59%) and oleic (32.7–54.9%) acids, as well as palmitic (9–11%) and stearic (5–10%) acids. Protein, nutrition, vitamins, calcium, and phosphorus are among the macro- and micronutritive values found in the crop. According to various research agencies, sesame oil may be utilized as a substitute for olive oil, and its by-products can be used as a protein supplement. When sesame oil is combined with other nutritious oils such as soybean or rice, it can provide a significant nutritional benefit. Sesame oil can help with neurological, dermatological, and cardiac issues.

Sesame oil's chemical structure, which includes a minimum concentration of saturated fatty acids (less than 15%) and the presence of antioxidants, has health-promoting effects in humans, including lowering cholesterol levels and hypertension, neuroprotective effects against hypoxia or brain damage, and lowering the incidence of certain cancers [27–30].

## 3. Summary and Conclusions

Sesame is a versatile crop that grows both cultivated and wild in Ethiopia, with a wide range of cultivated sesame varieties. It is one of the most widely planted oilseed crops in the country, accounting for 30% of the total output. Ethiopia's northern and northwestern areas, which border Sudan and Eritrea, produce most of the country's sesame oil crops. Sesame, the white gold crop, is the world's second most exported crop after coffee, accounting for 14% of total global exports. Ethiopia is one of the world's top six sesame producers. Sesame is one of the first oilseed crops, and it is grown in tropical and subtropical climes throughout Asia, Africa, and South America.

Its output and productivity are increasing year after year because of its economic importance, and it may produce a fair yield even in dryland regions. China, the Chinese mainland, the United Republic of Tanzania, Ethiopia, Uganda, Burkina Faso, and Myanmar produce the most kg per hectare of sesame in the world.

Similarly, sesame production in Ethiopia is increasing because of its importance in the lives of sesame-growing farmers and the rise in price with the increasing demand. However, there have been challenges on the supply and demand side of sesame oil crops. International price volatility, currency fluctuations, excessive speculators, illicit trade, constrained productivity levels, and local market pricing distortions, according to traders, are the key factors for the declining export trade performance. The significant difference between FOB (free on board) export pricing and local trade prices at ECX shows the local market price distortion.

Sesame oil is primarily used in the kitchen. Sesame oil can be found in soaps, paints, scents, medications, and pesticides. The cake made from the oil extracted from the seeds is a high-protein meal for poultry and ruminants.

Although there have been various challenges in a temporal and spatial context, sesame yields are predicted to rise in Ethiopia. A productive crop was aided by using improved seeds, additional inputs, and better farming systems such as row planting and improved agronomic methods. Those challenges of sesame production, such as drought, low sesame variety production, market volatility (domestic and world market), traditional production systems, and inadequate technology (planting; harvester), all combined make sesame productivity less than 10 qt/ha in most areas under farmer management. There needs to be more intensive production than utilizing a vast production system.

### 3.1. Possible Suggestions

It will be critical to establish modern sesame production systems in Ethiopia. This will require improved production and processing techniques, and the advancement of varieties with higher productivity potential and wider adaptability and the use of improved crop protection techniques, as well as capacity building by agronomic experts and enhanced soil moisture to stabilize crop growth and development across the sesame-growing areas, are vital issues to be considered to enhance its performance.

Moreover, since Ethiopian sesame is in high demand on the global market, hence more attention should be given to the crop by addressing the following issues:

- Financial institutions need to expand to fund sesame production and productivity.
- As can be verified from empirical studies, sesame crops can perform better under irrigation practice, so increased irrigation availability throughout the country is vital to becoming more visible in sesame production in the domestic and global market.
- Sesame seed production could be boosted by increasing the area under cultivation and using proper agronomic procedures and coupled with better varieties and technologies. Hence, farmers, governments, academics, and other concerned stakeholders must intervene to enhance sesame products and productivity.
- To this end, future research should focus on how to increase seed output in farmers' fields and reduce the yield gap between researchers and farmers while adhering to sound agronomic principles and appropriate technologies for sesame cultivation and processing.

## Figures and Tables

**Table 1:** Productivity of sesame in the world.

Countries	Productivity (kg/ha)
Myanmar	583.3
India	341.9
China, mainland	1312.5
Sudan (former)	260.5
United Republic of Tanzania	666.7
Ethiopia	661.3
Uganda	620.7
Nigeria	485.3
Mozambique	523.8
Burkina Faso	593.8

Source: http://faostat.fao.org/site/567/DesktopDefault.aspx?PageID=567#ancor.

**Table 2:** Sesame production by region in Ethiopia 2014/15.

Region	Area (ha)	Production (ton)	Productivity (ton/ha)
Tigray	1,20855.45	850,45.133	0.704
Amhara	1,69,988.58	1,122,09.218	0.66
Oromia	82,018.04	602,76.539	0.735
Benishangul-Gumuz	40,722,.60	278,093.345	0.68
SNNPR	6,365,70	3,165.097	0.497

Source: Abadi [1].

**Table 3:** Productivity of oil crops.

Oil crops	Productivity (t/ha)	
	2008	2013
*Noug*	0.56	0.772
Sesame	1.01	0.735
Linseed	1.12	0.920
Groundnut	1.11	1.402

Source: CSA [20].

**Table 4:** Estimated production volume of major oilseeds (metric tons).

Crop	2018/19	2019/20	Volume change	Percent change (%)
Sesame seed	300,000	340,000	40,000	13
Niger seed	300,000	305,000	5,000	2
Soybean	190,000	200,000	10,000	5
Total	790,000	845,000		7

Source: FAS Addis Ababa estimates.

**Table 5:** Sesame export destination.

Partner	Market share	
	Volume	Value
Israel	27.7%	27.3%
China	18.0%	17.9%
UAE	13.1%	13.5%
Vietnam	8.2%	8.0%
Japan	7.4%	7.8%
Singapore	7.0%	7.0%
Turkey	5.0%	4.8%
Saudi Arabia	3.5%	3.4%
Jordan	2.5%	2.5%
Yemen	1.8%	1.6%
Subtotal	94.2%	93.9%
Others	5.8%	6.1%
Grand total	100.0%	100.0%

Source: TDM.

**Table 6:** Comparison of the nutritive value of the food nexus with linseed, Niger, pumpkin, sunflower, and Ethiopian kale seeds.

Food and description	Local name	Food energy	Moisture	Nitrogen	Protein	Fat	Fiber	Ash	Calcium	Phosphorous	Iron
		Calories	%	Grams	Grams	Grams	Grams	Grams	Milligrams	Milligrams	Milligrams
Sesame seed, boiled	Selyit, qiqqil	324	49.2	2.33	14.6	26	5.8	2.3	482	427	11
Sesame seed, roasted	Selyit, yeteqwolla	662.5	0.2	4.01	25.3	56.1	7.1	4.3	816	631	25
Sesame seed, dried	Selyit, dereq	597	3.6	3.59	22.4	45.8	14.5	4.4	1445	459	10.8
Linseed, boiled	Telba, qiqqil	186.6	66.1	0.99	6.2	11	3.9	1	139	181	4
Linseed, roasted	Telba, yeteqwolla	620.2	0.9	2.66	16.3	47	9.8	2.8	304	483	8
Linseed, dried	Telba, dereq	510.9	5.8	3.2	17	29.7	10.3	3.6	227	454	29.4
Niger seed, boiled	Nug, qiqqil	334.1	73.2	1.92	12	23.7	10.9	2.9	260	514	12
Niger seed, roasted	Nug, yeteqwolla	589.6	1.4	3.19	19	42.8	16.4	4.7	472	856	12
Niger seed, dried	Nug, dereq	519.8	5.9	3.3	18.3	33.4	18.3	5.9	331	843	72.5
Pumpkin seed, boiled	Yedubba frye, qiqqil	288.5	51.7	2.39	14.9	20.5	9	1.8	26	456	6.5
Pumpkin seed, roasted	Yedubba frye, yeteqwolla	603.5	2.3	4.02	25.1	45.1	12.5	3.2	35	714	13
Pumpkin seed, dried	Yedubba frye, dereq	543.4	7.1	4	30	37.4	22.7	3.8	74	841	21.7
Ethiopian kale seed, boiled	Gomen zer, qiqqil	278.7	50.5	2.27	14.2	18.3	5.7	2.7	217	393	20
Ethiopian kale seed, roasted	Gomen zer, yeteqwolla	573.5	0.9	4.13	25.8	39.1	13	4.6	314	720	40
Ethiopian kale seed, dried	Gomen zer, dereq	517.4	6.1	3.9	4.5	35	7.8	8.3	368	594	392
Sunflower, *Helianthus annuus* L., boiled	Yeferenj suf, qiqqil	317.8	33.4	2.65	16.6	11	21.7	0.9	122	189	5
Sunflower seed, *Helianthus annuus* L., roasted	Yeferenj suf, yeteqwolla	559.8	1	2.57	16.1	33.8	33	1.3	179	265	6
Sunflower seed, *Carthamus tinctorius* L., dried	Suf, dereq	491.9	5.3	2.3	11.3	24.3	30.3	2.1	162	309	11.8

Source: Food and Agriculture Organization, N.D.

## Data Availability

The data used to support the findings of this study are included within the article.
